# Sonification of Complex Spectral Structures

**DOI:** 10.3389/fnins.2022.832265

**Published:** 2022-03-10

**Authors:** Mattias Sköld, Roberto Bresin

**Affiliations:** ^1^Composition, Conducting and Music Theory, KMH Royal College of Music, Stockholm, Sweden; ^2^Sound and Music Computing, KTH Royal Institute of Technology, Stockholm, Sweden

**Keywords:** sonification, complex spectral structure, music, spectrum, perception, listening, comprehension

## Abstract

In this article, we present our work on the sonification of notated complex spectral structures. It is part of a larger research project about the design of a new notation system for representing sound-based musical structures. Complex spectral structures are notated with special symbols in the scores, which can be digitally rendered so that the user can hear key aspects of what has been notated. This hearing of the notated data is significantly different from reading the same data, and reveals the complexity hidden in its simplified notation. The digitally played score is not the music itself but can provide essential information about the music in ways that can only be obtained in sounding form. The playback needs to be designed so that the user can make relevant sonic readings of the sonified data. The sound notation system used here is an adaptation of Thoresen and Hedman's spectromorphological analysis notation. Symbols originally developed by Lasse Thoresen from Pierre Schaeffer's typo-morphology have in this system been adapted to display measurable spectral features of timbrel structure for the composition and transcription of sound-based musical structures. Spectrum category symbols are placed over a spectral grand-staff that combines indications of pitch and frequency values for the combined display of music related to pitch-based and spectral values. Spectral features of a musical structure such as spectral width and density are represented as graphical symbols and sonically rendered. In perceptual experiments we have verified that users can identify spectral notation parameters based on their sonification. This confirms the main principle of sonification that is that the data/dimensions relations in one domain, in our case notated representation of spectral features, are transformed in perceived relations in the audio domain, and back.

## 1. Introduction

The work and ideas presented here relate to an ongoing research project concerning the development of sound notation from Thoresen's spectromorphological analysis symbols (Thoresen and Hedman, 2007; Sköld, 2020). The motivation for this research is how the lack of a standardized notation language for sound-based music (music not only relying on pitch structures) means that certain practices in music composition, performance, and music theory are not available for this kind of music. Thanks to traditional music notation's relevance for both perception and performance, a melody can be notated, performed and then re-transcribed from its performance. There is a tradition of notating electroacoustic music, but with representation specific to individual works (Karkoschka, 1972), see, e.g., Messiaen's *Timbres-Dures* (Battier, 2015) and Stockhausen's *Studie II* (Stockhausen, 1956). The main target groups for this research are composers, performers and musicologists, though we believe this kind of notation can be of use in other areas where the symbolic representation of sound structures are needed.

Having focused mainly on the design of the notation system itself, we are now considering the functionalities of software playback of the notated data. The idea is to introduce a workflow similar to what composers have come to expect from computer-assisted composition where algorithms result in data to be represented as musical notation. When working with pitch-based musical structures, composers can generate MIDI data in music programming languages for import into notation software for editing and printing. Though an old format, MIDI has remained the standard for rationalizing instrument instructions in software. In this text, we are presenting a framework for working with playback of sound notation as sonification of notated data, using a data format similar to MIDI acting as a bridge between notation and sound production. We are furthermore showing through listening tests that the notated data for four timbre-related notation parameters can be identified based on their sonification.

## 2. Background

Music notation made with computers started with Leland Smith's SCORE (Smith, 1972), a data format for computer music from 1967, developed to print graphical scores in the early 70s (Smith, 1973). During the 1980s, Mark of the Unicorn were influential in music notation on computers with their Composer's Mosaic and Performer (Belkin, 1993) before the introduction of Finale in 1988 and Sibelius five years later. The latter two would dominate the market for over a decade (Strawn and Shockley, 2014) and are still widely used.

Playback was part of the history of music notation software from the start Smith's SCORE was initially an inter-face for generating computer music in Mathews' MUSIC V in a data format tailored for composers (Smith, 1972). Following the establishment of the MIDI standard in the 1980s, MIDI playback would become an integral part of notation and music production software. Playback quality has been subject to much development over the years and a selling point for the software companies. Yet, digital playback of notation can be misleading for young composers orchestrating their work (Deutsch, 2016), and composition teachers warn their students not to rely on it in their work. However, we think it can be valuable if one appreciates it for what is—the sonification of the notation data.

## 3. Music and Sonification

As Carla Scaletti wrote in her overview chapter about data sonification, “sonification is not music,” but “music is sonification” (Scaletti, 2018). The latter is also our position in this article. For example, we consider the performance of a music score to be its sonification: different performances of the same score by different players with different instruments still acoustically communicate the same content, i.e., the relationships between the data written on the score (e.g., notes) are kept in the acoustics domain. Music has been used in a few sonification applications in the past. Musical sonification has been applied in movement rehabilitation (Kantan et al., 2021), in rehabilitation after stroke (Nikmaram et al., 2019), in gate retraining (Lorenzoni et al., 2018), and in rehabilitation of Parkinsonian dysgraphia (Véron-Delor et al., 2018). Music has been also applied for the sonification of visual artworks (Adhitya and Kuuskankare, 2012), for inter-active sonification of drummers' gestures for personalizing the resulting performance (Wolf and Fiebrink, 2019), and for the sonification of climate temperature data mapping them C major scale notes played by string instruments (George et al., 2017). Also, the spectrum of the light emitted by galaxies has been sonified by using harmonic sequences (Ballora, 2014).

The use of music in sonification applications can help users in understanding the underlying relationship in the data being sonified. Certainly, most of the users of sonification applications are more familiar with music and how it can communicate content such as musical structures and emotions auditorily rendered through recognizable sound sources (such as acoustical musical instruments) than with sonifications based on synthesized abstract sounds which can be difficult for a layperson to associate to a familiar sound source. Difficulty in recognizing a sound source can lead to problems in the understanding of the data to be communicated with sonification. In fact results from a previous study by Caramiaux and colleagues (Caramiaux et al., 2014) have shown how a listener's perceptual embodiment of a sound differs depending on if the action that has produced that sound can be identified or not: causal sounds were associated to the gestures that generated them, while non-causal sounds were represented with gestures associated to their acoustic properties.

In the study presented, in this article, we wanted to investigate how a complex sound property such as timbre can be correctly associated with its corresponding symbolic notation, while taking into account the sound's physical characteristics (spectral properties). Our hypothesis is that the sonic rendering of a complex score notation corresponds to its sonification, since the relationships found in the graphical representation in the written score are kept and recognized in the corresponding sound.

### 3.1. Notation Playback as Sonification

Playback of scores in music notation software can function as the sonification of data from which the user can hear key aspects of what has been notated. This hearing of data is significantly different from reading the same data. The digitally played score is not the music but can provide essential information about the music in ways that can only be obtained in sounding form. In his article on composition pedagogy, Daniel Deutsch notes:

Notation software playback is often misleading when it comes to timbre, texture, dynamics, and orchestration. However, it can be extremely helpful in assessing the overall contour and structure of a composition (Deutsch, 2016, p. 57).

If one were to assume that the software playback should approximate the perception of the music as performed, there are indeed problems. However, Deutsch is right that the playback is a great help for assessing the composed structure. This is because all notated parameters are symbols of musical values for which time is an essential aspect. As an example, when sonifying a given pitch structure, its pitches are no longer experienced in terms of left and right, up and down. Instead the pitches are heard as parallel streams in terms of then and now. But like medical staff must learn to interpret the sonified data of their monitoring equipment, the software user must learn to interpret the playback rather than assume it to be an approximation of a musical performance. For composers using notation software it is, therefore, essential to consider what data is being sonified and how it informs the musical structure as a whole. There is also a learning process involved in acquiring an understanding of how sonified structures change as they are performed live.

### 3.2. Sonification of Traditional Music Notation

There is a long tradition of *simulating* scores. Before the computer, and still today, composers use the piano to assess musical ideas written for other instruments. With the development of software tools for notation came the possibility for digital simulations of scores not limited to a piano's or a pianist's capabilities. As these digital tools improved, the simulations became more realistic, to the extent that they could replace the actual performance. For the scoring composer, this is not necessarily the goal—the playback of notated data is rather used for providing a time-dependent understanding of the notation. It is then important to consider that different musical parameters in traditional notation have different preconditions for providing relevant information through sonification:

- Sonification of melodies and harmonic structures can be successful provided that the notation is played using sounds with stable pitch. If the notation playback is performed with different sounds for different voices, the harmonic structure is easier to comprehend if the sounds have equal amplitudes and preferably also similar timbre. There is a risk of misleading sonification of the harmonic structure if the sonified sounds are very short, since the actual instruments meant to perform the score may need more time to establish a stable pitch. This can also vary over the register of one and the same musical instrument.
- Sonification of the notated rhythm can to some extent provide relevant information provided that the sounds used for sonification have the same energy articulation and amplitudes volume as the ones referred to in the score. When performed, rhythm is often subject to continuous variation and genre-specific rules that can be difficult to imitate without much editing of the sonified output.
- Sonification of dynamics can be very difficult for scores aimed for acoustic live performance, since amplitudes of the individual instruments in relation to one another are constantly negotiated between the performers. However, overall structural dynamic changes can be sonified to provide information on the experience of form as expressed through dynamics development.
- Orchestration is a music phenomenon considered hard to represent faithfully through notation software playback (see, e.g., the quote from Deutsch above). It can, however, be of great help since the result of blending timbres can be hard to predict. A good example is the widely used Hauptwerk system,^1^ a virtual pipe organ with a massive archive of sampled organ stops that can be combined to simulate organ registration without sitting in front of the actual instrument.

A general problem with digital sonification of scores for acoustic performance with regard to orchestration and dynamics has to do with the positions of the performers. In a full orchestra there are significant distances between the instruments, as problematized by Skålevik (2007). Besides directional hearing, these distances heavily affect dynamics and the blending of timbres.

## 4. The Sound Notation System

**Figure 1:**
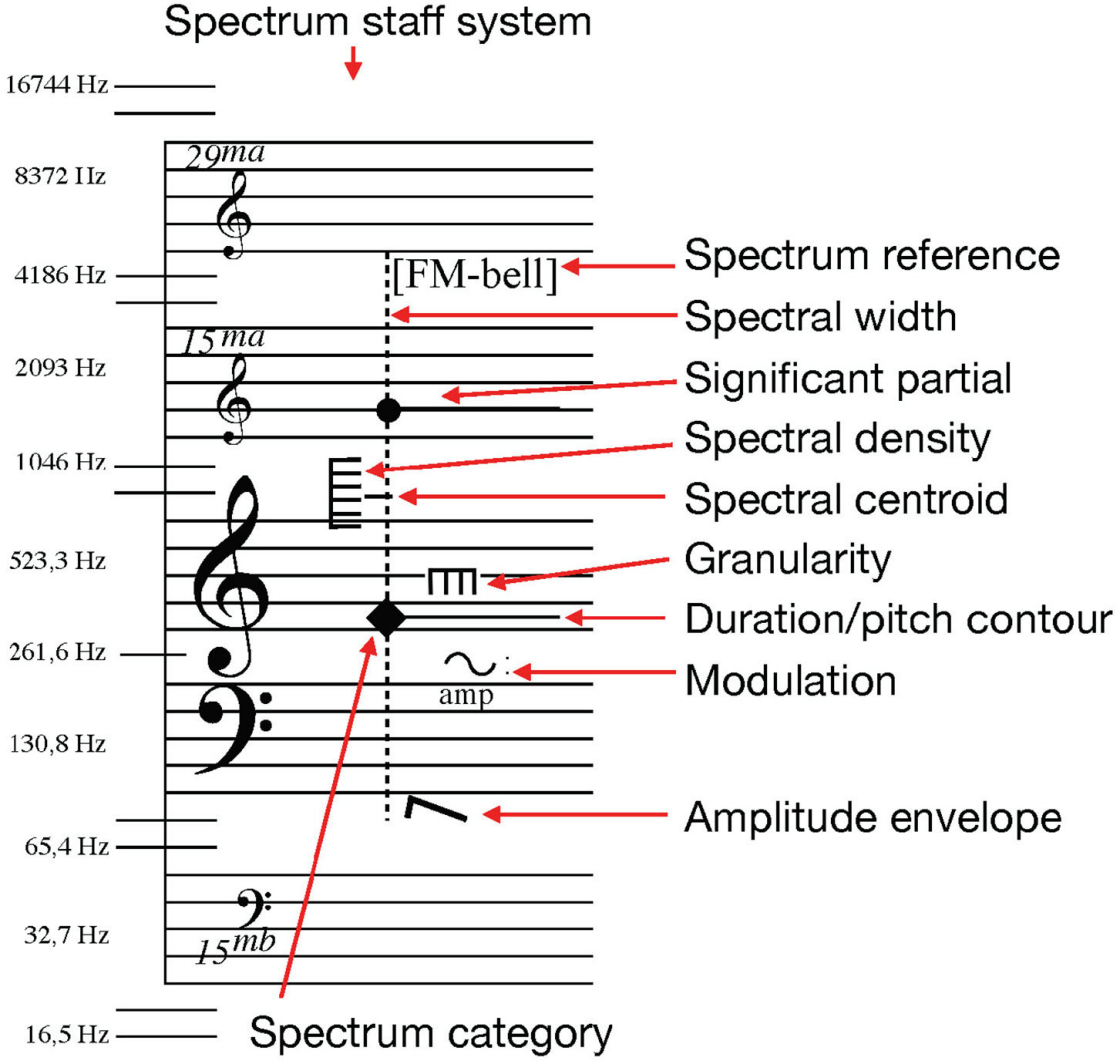
An overview of the basic notation features of the sound notation system, used to describe one dystonic (inharmonic) sound object with the partial with the highest amplitude at F4, and a secondary high-amplitude partial at G6. There is significant spectral information, spectral width, between 80 Hz and 4.7 kHz, and it is a nearly maximum density spectrum as indicated by the vertical comb with six teeth. This image has been previously published (Sköld, 2020).

The sound notation system proposed here is an adaptation of Thoresen and Hedman's spectromorphological analysis notation (Thoresen and Hedman, 2007; Sköld, 2020). Symbols originally developed by Thoresen from Schaeffer's typo-morphology (Schaeffer, 2017) have in this system been adapted to display measurable spectral features of timbre structures for the composition and transcription of sound-based musical structures. Spectrum category symbols are placed over a spectral grand-staff that combines indications of pitch and frequency values for the combined display of music related to pitch-based and spectral values (see Figure 1). Though pitch and frequency are not inter-changeable concepts they coexist here in the same manner as they do in computer music environments^2^.

## 5. MIDI for Playback

Sonification of traditional notation data is commonly made using MIDI. Connecting notation and MIDI data is logical considering that both carry instructions for musical instruments. In DAWs like Logic Pro one can choose to view the same data as an MIDI event list, a piano roll, or as score. Major notation software can import and convert MIDI data into notation as well as export the notation as MIDI. In both the import and export cases, editing is needed to compensate for the incompatibility of data which mainly have to do with their different purposes: notation as instructions to a performer and MIDI as instructions for a (keyboard) instrument. MIDI has been important for algorithmic composition to the extent that the limitations of MIDI may have to do with what musical parameters are subject to the algorithmic work (Nierhaus, 2009). A strength of the connection between notation and MIDI is that composers with knowledge of MIDI can tweak the notation playback, editing the MIDI data directly. There has been suggestions of modifications of the MIDI standard to accommodate notated features, such as the difference between a C sharp and a D flat (Hewlett, 1997). Because of the inadequacies if MIDI, OSC (Open Sound Control)^3^ has been considered its successor but has failed yet to replace MIDI as the standard for music communication, and OSC comes with its own problems (Fraietta, 2008).

## 6. New Data Format for Playback

Our solution for sonifying the sound notation system was to define a data format similar to basic MIDI instructions that could correspond to the new notated symbols as well as be used as instructions for sound synthesis. In order for the new data format to be successful in algorithmic composition, it needs to inherit these properties from the MIDI standard: (1) it should be comprehensible enough so that composers can work with the data understanding what the data represents and (2) the instructions should be fairly easy to use as instructions for synthesis.

### 6.1. Sound Object Components Through Different Channels

Musical events in the MIDI standard are closely related to the idea of the piano keyboard (Moog, 1986). Even if a pitchbend message may introduce changes to a sound, its identity is tied to all keys pressed on a particular channel. This design works for keyboard-controlled sequencing but limits the flexibility of MIDI when used for experimental composition.

For our data format, we keep the basic MIDI structure of sounds being turned on and off on different channels, but rather than associating sound object components with keyboard keys, we use arbitrarily assigned ID numbers for keeping track of which components are active. Like MIDI notes, several sound object components can be active on one channel. This channel assignment is typically used to place multiple components from a composite sound object on the same (grand) staff system.

### 6.2. Beginning and Ending Sound Components

Central to the data format are the *begin* (B), *end* (E), and *attack* (A) messages. B begins a new sound object component, with a given spectrum category to determine its fundamental spectral characteristics and a pitch value in midicents to set its fundamental, its most significant partial, or its spectral centroid depending on the category. E ends the component, marking the termination of the amplitude envelope. A is similar to the B message but is used to specify the characteristics of the attack of a sound. The difference is that A terminates automatically and does not need an E message to end.

### 6.3. Spectral Characteristics

Besides the spectrum category, set when beginning the sound object component, there are specific messages to set and change the spectral characteristics of the component. W sets *spectral width*, which is the bandwidth of the sound. Spectral width is set using two frequency values, the lower and higher limits of the frequency range. A zero value means that this value is set to the same frequency as the pitch contour. This is practical for harmonic spectra where the lower value is commonly (though not necessarily) the same as the fundamental of the spectrum.

*Spectral centroid* (C) is set with a single frequency as argument to set the center of the spectrum's spectral energy, *spectral density* (D) is set as a float value between 0.0 and 1.0 which when notated are interpreted as discrete vertical comb-like symbols. *Spectrum reference* (R) takes a text strings as argument to indicate similarities to any named sound source.

### 6.4. Energy Articulation and Variation

The *amplitude envelope* (EN/ES) of a sound is indicated using either EN (ENvelope) for an ADSR envelope or ES (Envelope Shape) for predefined envelope shapes ranging from -1.0 to 1.0 where 1.0 produces a percussive envelope, 0 produces a flat organ style envelop while -1.0 is the reverse of 1.0, which means slow attack and fast release. *Variation* (V/VM) is a generic function (cf. MIDI control change messages) for introducing changes to a named target using values between 0.0 and 1.0. The target can be anything. V is used for introducing continuous value changes while VM denotes variation as cyclic modulation invoking a given waveform, such as sine or sawtooth. *Granularity* (G) refers to sounds made up of sequences of short sound grains, and it is set terms of velocity and coarseness as float values, which in the notation are displayed as discrete vertical comb-like symbols.

### 6.5. Display and Playback of Changing Values

In both notation and playback, value changes can be interpreted as breakpoints or instant value changes similar to how MIDI control change messages work. The former requires less data to create smooth interpolating value changes while the latter is necessary when working in real-time.

The first argument for any message is the time indicator. This can be formatted differently depending on the purpose of the notation. For the initial tests, time is indicated as elapsed time in milliseconds.

## 7. Software Implementation

**Figure 2:**
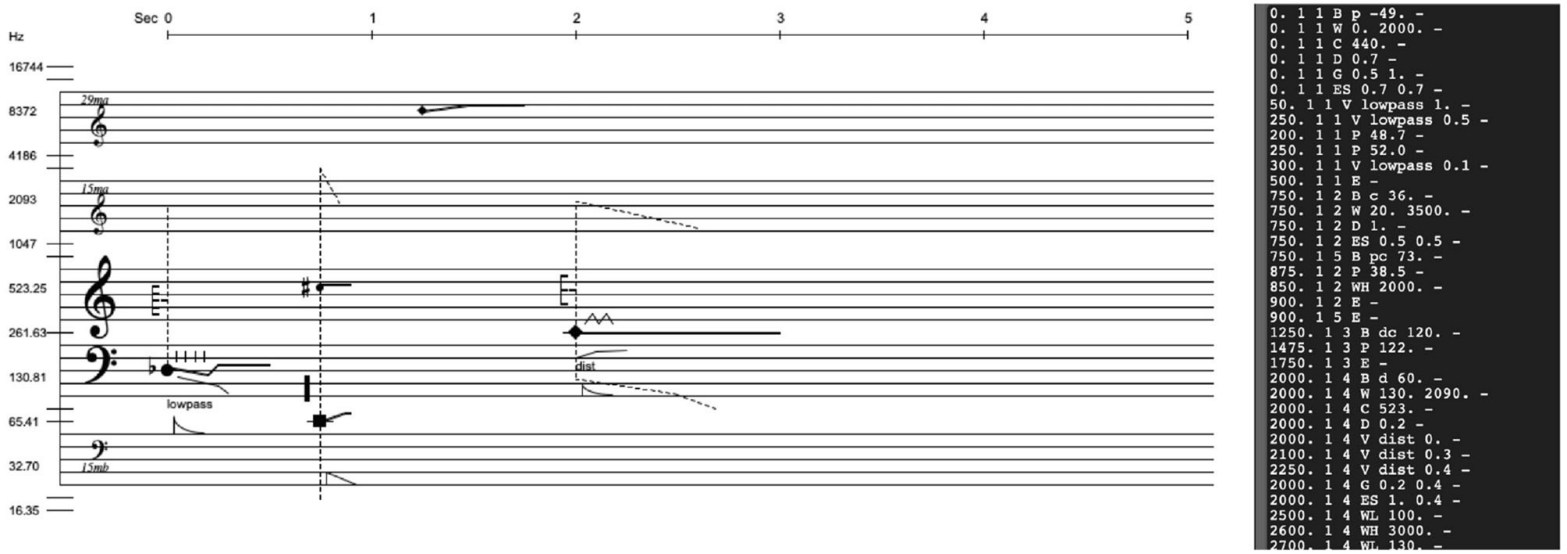
Screen shot of the max implementation of the sound notation, in which the data in the black text block to the right has generated this score excerpt.

A prototype of the software implementation of the notation was made as a Max patch^4^, in which the notation is displayed and sonified by entering data messages in the format described above. Figure 2 is a screenshot from the patch, with the data input in a text editor to the right and the resulting notated symbols on the left.

### 7.1. Sound Design

Without any tweaking, the sound of the sonification should be rather generic, acting as data representation of the spectral parameters of the notation. When specific music parameters are to be evaluated, realistic simulations of instruments can stand in the way of hearing and understanding the data one is interested in. This is because one aim of the project is to enable composition with timbre parameters regardless of specific sound sources. (And even if one has particular sound sources in mind, simulations of instruments can be misleading due to the different conditions for different acoustic performances). Basic waveforms from synthesizers such as sine and sawtooth waves are not suitable either to act as generic sounds since they represent extreme cases of spectral characteristics. Since the notation makes possible independent changes to spectral parameters on a sound object, additive synthesis was considered the most convenient method for the sonification.

The software implementation of the playback was centered around the interpolating oscillator bank in Max, *ioscbank*~^5^, which can produce additive synthesis from an inter-leaved list of frequencies and amplitudes. The synthesis process regarding spectral properties is as follows:

- The spectrum category and its pitch values determine the logic for how partials are positioned along the frequency range to produce a list of partials along the frequency axis, either as multiples of a root frequency or at random frequencies values. Initial amplitude values are also generated for all partials.
- The spectral density value affects the distribution of amplitude values.
- The spectral width low and high values affect the amplitudes along the frequency axis with effects similar how highpass and lowpass filters affect audio.

**Figure 3:**
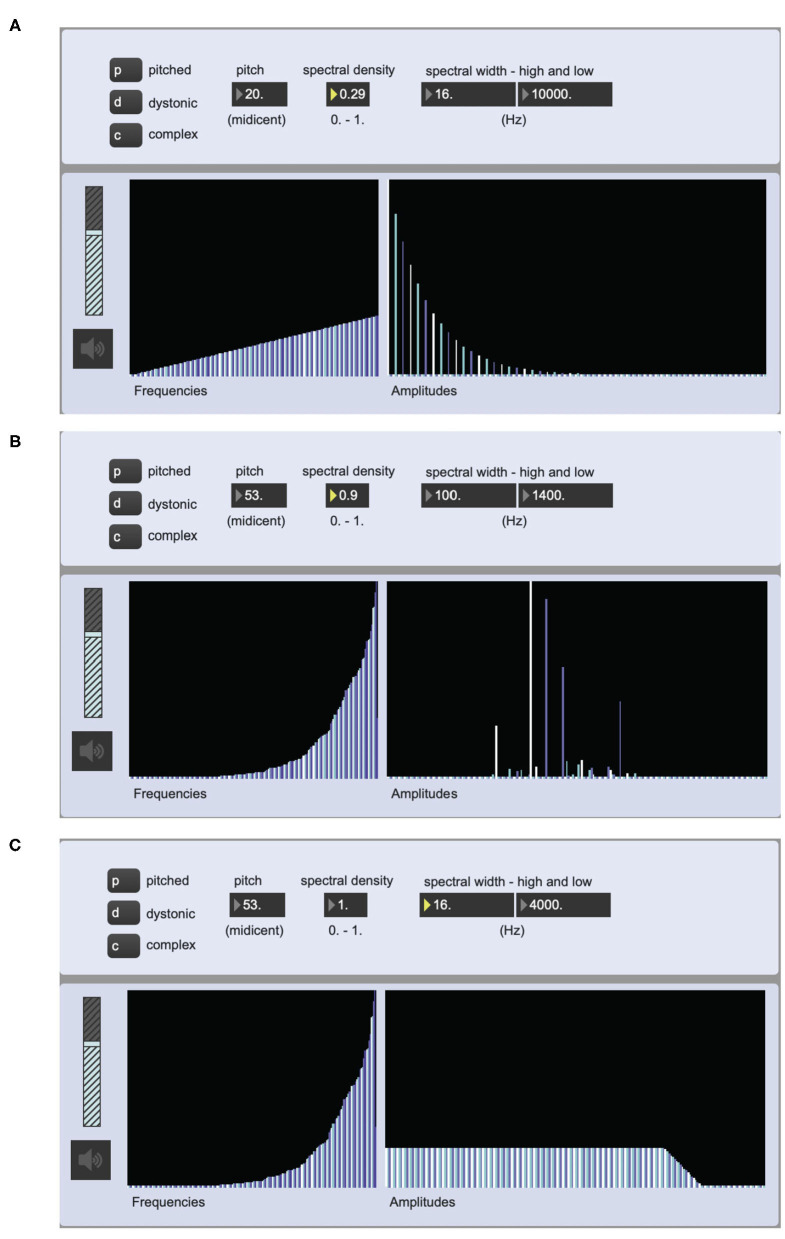
Screen shot of a Max prototype for the implementation of the sonification of the notation data. The multisliders (interactive tables) show the partials of the oscillator bank along their *X*-axes and the corresponding frequency values (16 Hz-17 kHz) to the left and amplidudes to the right (0-1). The different colors of the bars are there to differentiate between adjacent partials. **(A)** is set to play a pitched sound object with a harmonic spectrum. **(B)** is set to play a dystonic sound object with an inharmonic spectrum, while **(C)** is set to play a complex sound object—in this case a noise.

Figure 3 shows three examples of the graphical user inter-face used for testing the sound synthesis engine to be used for sonifying the spectral properties of the notation.

To compensate for the low density of the harmonic spectra produced using the oscillator bank, pitched sounds where mixed with filtered sawtooth waves. And to guarantee the correct spectral width, low and high pass filters were part of the signal chain of the sonification process. It should also be noted that despite the fact that the sonified parameters have to do with spectral characteristics of the sound, there is great room for variation when sonifying any given notation example, as is the case with traditional notation.

## 8. Evaluation

In order to evaluate the notation playback as a means for sonification of the notated data, listening tests were conducted. The first was a longer test with multiple choice questions, conducted using a web survey. This was sent to specific participants who are musicians, composers and music academics. To shed light on the survey results a small version of the test was conducted with four participants in the same room, answering six of the questions from the survey, freehand-drawing their notation symbols on printed staff systems. This smaller session also included discussions about the reasoning behind the answers.

### 8.1. Participants

For the web survey, 36 people completed the test (6F, 12M, 3NB, 15 unknown/did not want to answer; average age 37.31, SD=9.84). For the smaller in-depth study, four students with electroacoustic music composition as their main subject participated. Only listeners with some musical training were asked to participate in the tests since the capacity for interpreting symbols related to staff-notation and a frequency scale is subject to musical training.

In the web survey, participants were asked about age, gender, and nationality. Nationality was included because of possible culturally defined differences in the perception of music, as explored by Pembrook (1997) and Trehub et al. (2015). They were also asked to describe their music reading skills (traditional Western music notation) and their music technology and/or electroacoustic music proficiency. Both skills had three possible answers: none, some skills and professional. Finally, they were asked to specify their listening conditions for the survey, including audio equipment used.

### 8.2. Test Design

All test images and sound files can be accessed at https://doi.org/10.5281/zenodo.5831225. The multiple-choice test contained 16 questions, each with four alternative answers labeled A through D. Since testing the full capacity of the notation system would be impractical, four parameters related to the notation of timbre were selected for testing. Each of the following parameters were tested with four questions:

- Spectral category
- Higher limit of spectral width
- Lower limit of spectral width
- Spectral density

**Figure 4:**
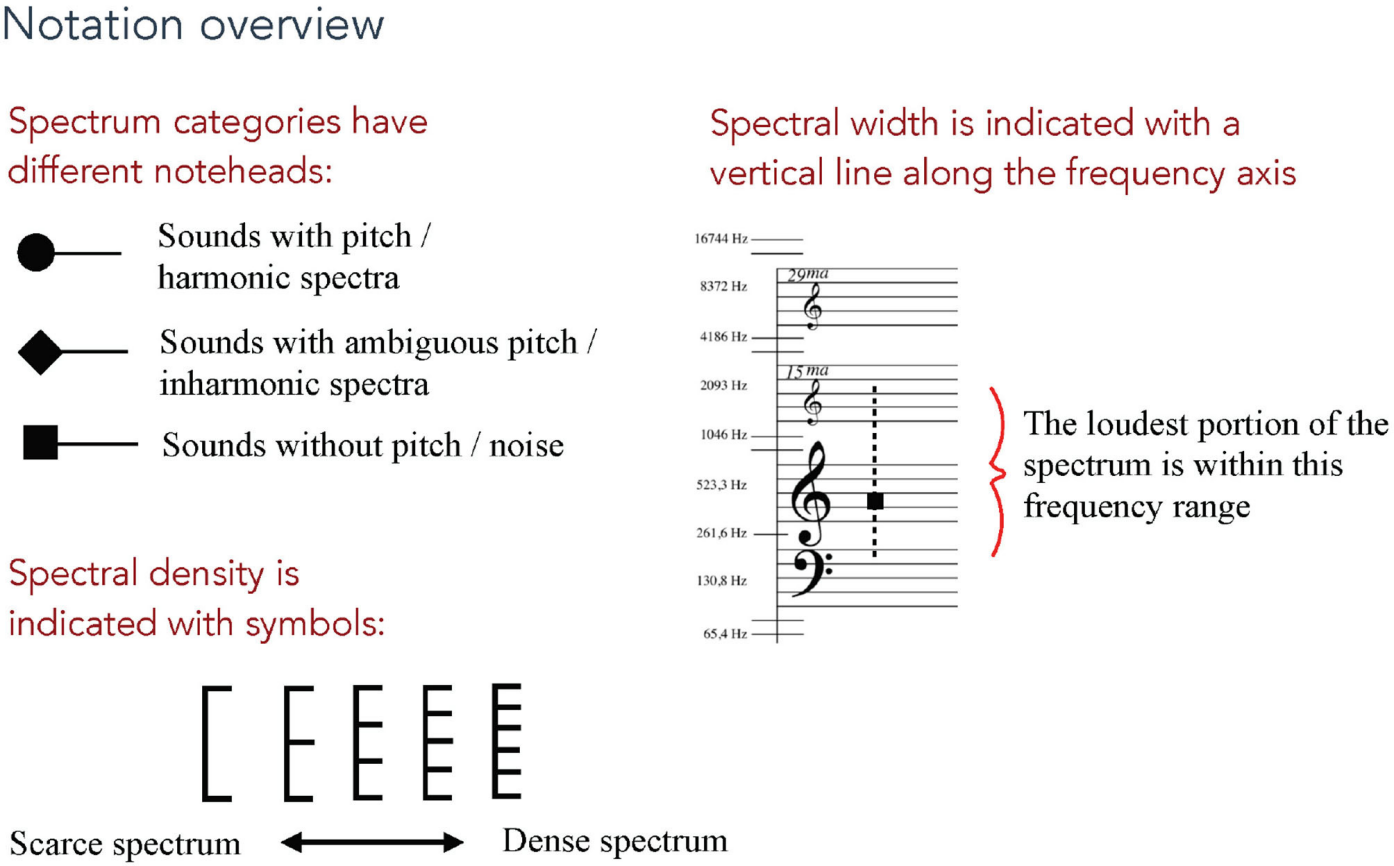
Overview of the notation features in focus in the listening tests. The three parts of this image were presented separately in the beginning of the multiple-choice test. And this image was also available as a PDF download for participants to use as a reference throughout the test.

These parameters were selected because they represent aspects of the sound not commonly included in traditional music notation. Spectral centroid is also an important parameter for timbre perception but it was not included in these tests because of how it overlaps with the combined information from the spectral category and the spectral width, particularly for complex spectra where the pitch position of the (square shaped) category symbol is the spectral centroid. An overview of the notation symbols for these features is shown in Figure 4. *Spectral category* defines the most fundamental aspect of a sound's spectrum–if it is mainly harmonic, inharmonic or non-harmonic (noise). The higher and lower limits of *spectral width* delimit the most significant range of a sound's spectral energy. (These limits are defined as the highest and lowest limits where the spectral energy drops below -12 dB in relation to the sound's amplitude peak). *Spectral density* is a relative measurement of the amount of partials with high amplitudes within a given spectral width. For its sonification, spectral density behaves differently for different spectral categories. E.g., the possible frequency values of partials in a harmonic spectrum are fixed in relation to the root frequency, so there is a limit to how densely the partials can be positioned. It was important to define these spectral parameters in as general terms as possible, and for the sake of clarity they are represented by one value per sound object in the test questions. In the analysis of acoustic sounds, one would take into account how the parameters change over time.

For the small in depth listening test six randomly selected questions from the web survey were used, without the multiple choices. Instead, printed empty grand-staff systems with a frequency axis on the left side were provided for the participants to draw their symbols on. The questions were identical to the ones used for the multiple choice test, but without any notation examples to choose from. The questions used were a random selection from the 16 in the survey: #4, #7, #9, #12, #14, and #15. As can be seen in Table 1, these questions cover all four parameters in focus.

**Table 1:** Table of results from the multiple choice notation survey.

**Parameter**	**Question**	**Correct answer**	**Correct pattern/contour**
Spectral category	1	94%	100%
	2	86%	97%
	3	86%	92%
	4	92%	97%
	Mean:	90%	97%
Spectral width lower limit	5	72%	97%
	6	58%	97%
	7	61%	78%
	8	61%	94%
	Mean:	63%	92%
Spectral width higher limit	9	78%	97%
	10	86%	97%
	11	81%	97%
	12	81%	100%
	Mean:	81%	98%
Spectral density	13	64%	64%
	14	94%	94%
	15	92%	100%
	16	94%	94%
	Mean:	86%	88%

### 8.3. Listening Test With Multiple Choice

Before answering the main questions, the test participants were presented with a brief overview of the three notation symbols used in the test and what they represent. These symbols were presented on separate pages so as not to confuse the participants. There was also the possibility to open a single-page overview image of these notation parameters in a separate tab or window in one's browser–this overview is shown in Figure 4.

**Figure 5:**
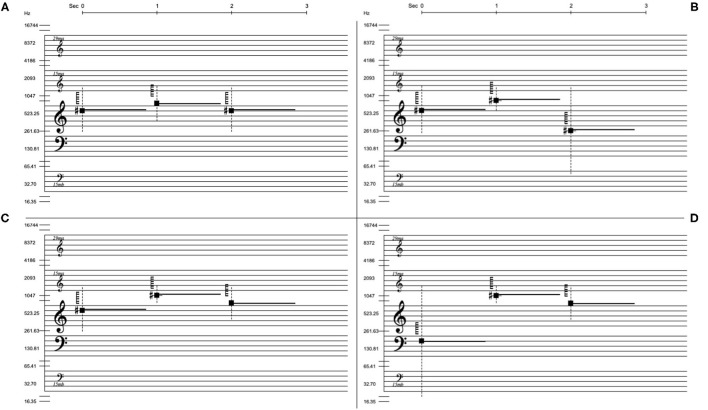
The four notation choices for question #5 of the multiple choice listening text. For this question, lower limit of spectral width is in focus, indicated by the lower end of the dashed vertical line. **(C)** is the correct answer and it shares its overall frequency contour with **(D)**. **(A)** and **(B)** are incorrect but are still fairly similar to **(C)** and **(D)**.

Each of the 16 questions involved a three-second sound file and an image with four alternative notations of three-note phrases as shown in Figure 5. The sound file, which one could listen to multiple times, contained the sonification of one three-note phrase. In the image of four alternative notations, one was the correct sonified notation, and the three others were similar but incorrect notations concerning the parameter in focus. These incorrect phrases varied in how much they deviated from the correct notation. The instruction was: “Play and listen to the sound example as many times as you like and choose the notation you believe best describes the sounds you heard.” In order to be able to assess the sonification of the notated parameters separately, each group of four questions had one test parameter changing and the others remaining the same. The questions were presented in a randomized order to each participant.

We wanted to know whether participants could distinguish the correct contour of the parameter changes in focus, but also if they could perceive the actual parameter values of the sounds and relate them to the notation symbols. Therefore, all questions except #13, #14, and #16 had one incorrect answer with a contour or pattern similar to the correct answer: In question 1 the correct sequence of spectrum categories is P P D (two pitched and one dystonic). The other possible choices are P D P, D D P, and D P D. So the D D P choice shares the same pattern as the correct answer, but with the wrong spectral categories. Similarly, question 5 (see Figure 5) has the correct sequence of spectral width lower limit: 250 800 400 Hz, with the wrong answers displayed as 20 800 400 Hz, 250 400 250 Hz, and 250 600 50 Hz. A correct interpretation of the sonified notation requires the listener to identify where there is energy along the frequency axis. The 20 800 400 Hz alternative has a contour similar to the sonified notation but indicates lower frequency content for the first note. It is worth noting that an incorrect answer, particularly concerning the perception of low frequency energy, can arise as a result of poor listening conditions.

So for all but three questions there was a 50% probability of selecting the correct contour or pattern and a 25% chance of selecting the correct answer. And for the remaining three questions (#13, #14, and #16) there was a 25% chance of selecting the correct answer, as well as the correct contour.

### 8.4. In Depth Listening Test

The in depth listening test was conducted with four composition students. A randomized selection of the sonified notation from the online survey was used, presented to the group through loudspeakers in the room. Only six questions were used. Instead of multiple choices, the students had printed grand-staff-systems on which they were asked to draw the notation of what they heard. The randomisation resulted in this selection and order: Questions #9, #4, #12, #7, #15 and #14. This included examples with all four parameters in focus.

The students had worked with the sound notation system before in a course module on Sexology (KMH, 2019) but were presented with printed copies of the same notation overview as in the multiple-choice survey to use for reference. Mattias Skld performed the test, playing each sonification example multiple times. When participants asked for another playback of the sound, this was done. Since there was no instrument or tuning fork available for reference, a reference recording of the C4 on a piano was played before each new question. Otherwise, the students would need perfect pitch to place the symbols correctly.

Since the participants were not told what parameter was in focus they had to listen for changes in all four parameters. After concluding the experiment with six questions, we had an open discussion regarding the results.

## 9. Results

### 9.1. Listening Test With Multiple Choices

Table 1 shows the results from the multiple choice notation survey divided into the four notation parameters in focus. The table shows percentage of correct answers per question and as an average per notation parameter. The table also shows the percentage of all answers with the correct pattern or contour of the parameter in focus. This value also includes the correct answers.

For recognizing contours or patterns of parameter changes (the rightmost column), the questions for the four parameters averaged equal to or more than 88% correct identifications of patterns/contours, well over the 50% probability for guessing. For identifying the correct parameter values (the middle right column), spectral category and spectral density were identified by 90 and 86%, respectively, while the lower and higher limits of spectral width provided lower results (63 and 81% on average), but all well over the 25% probability for guessing. The mean score of correct answers for all participants in all four parameter categories was 80%.

Two-sample *t*-tests assuming unequal variances were performed to compare the mean values of correct results in relation to gender and nationality (we had this information from 22 participants). There was not a significant difference in amount of correct answers between male participants (*M* = 12.75, *SD* = 1.96) and female participants (*M* = 12.5, *SD* = 1.52); *t*(16) = 2.16, *p* = 0.77. There was neither a significant difference in amount of correct answers between Swedish participants (*M* = 12.69, *SD* = 1.5) and non-Swedish participants (*M* = 12.40, *SD* = 1.67); *t*(19) = 2.31, *p* = 0.76. Comparing the test performance between age groups 20-29, 30-39, 40-49, and 50-59 did not show any significant differences, *F*(3, 32) = 0.58, *p* = 0.63.

Table 2 shows the impact of musical training and listening conditions on the participants' average amounts of correct answers. Musical training did not affect the results much, though small differences indicate that proficiency in music technology and/or electroacoustic music had a positive impact, while participants who stated that they have some skill of music reading, handled the test somewhat better then they who stated they had a professional level. The one important factor impairing the results were the listening conditions, where medium and poor quality correlated with much worse performance for value changes of the lower limit of spectral width. In other words, changes of low frequencies were not perceived under worse listening conditions.

**Table 2:** Amount of correct answers related to participant information.

**Parameter:**		**Category**	**Width low**	**Width high**	**Density**
Listening	High quality	88%	71%	83%	87%
	Medium quality	89%	63%	80%	86%
	Poor quality	100%	13%	88%	88%
Music technology	Professional	92%	63%	82%	87%
	Some skills	87%	63%	81%	85%
Music reading	Professional	87%	58%	81%	87%
	Some skills	95%	75%	82%	84%

*The question on music technology asked about the proficiency level regarding music technology and/or electroacoustic music*.

### 9.2. In Depth Listening Test

**Figure 6:**
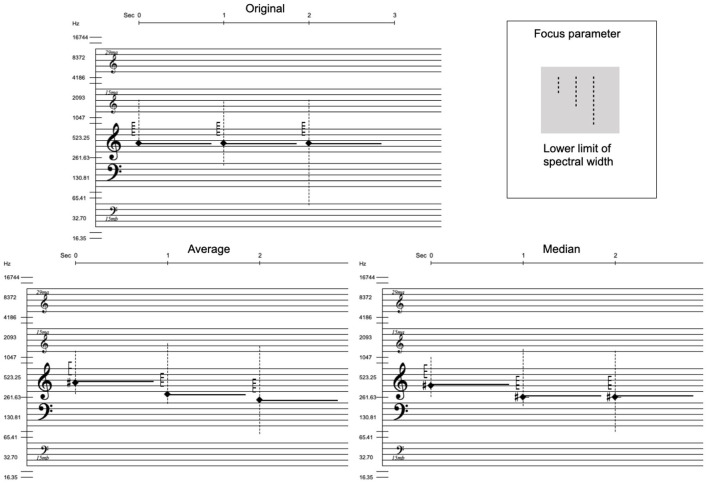
Comparison of the original sonified notation and notation generated from the average and median data retrieved from the hand drawn notation. This is question 7 in the original set of 16 questions, and the parameter in focus is the lower limit of spectral width.

The main data from the small in-depth study was four sets of six hand drawn transcriptions of our sonification. For two questions (#9 and #15) only three participants drew symbols for the parameter in focus. In order to assess the data and maintain participant anonymity, the hand drawn notation was converted into numerical data (except the spectral category) in the data format described above. New notation images were generated from the average and median values of the participants notation data making it possible to visually compare their shared result with the original sonified notation. Figure 6 shows this generated notation for question 7, comparing the original with the combined results from the study. Table 3 shows these value comparisons for five of the six questions, which have different parameter values in focus. (Question #4 had spectral categories in focus and was, therefore, not suitable for numerical analysis.) Since the spectral width values were drawn along a logarithmic axis over a staff system, mean and SD values were calculated on midicent^6^ values and then converted to Hertz.

**Table 3:** Table of the in-depth test results for five of the six randomly selected questions, and to the left are the original three values for the parameter in focus for each question.

**#**	**Parm**	**Original**			**Test mean (SD)**			**Test median**		
7	WL	400	200	50	290 (151)	211 (82)	78 (37)	269	191	92
9	WH	1600	800	400	2744 (3590)	1544 (1251)	788 (809)	4200	2000	880
12	WH	750	2000	5000	650 (392)	1612 (1439)	2527 (3517)	523	1480	2096
14	D	0.8	0.2	0.2	0.4 (0.34)	0.2 (0.1)	0.1 (0.12)	0.3	0.2	0.1
15	D	0.2	0.6	0.6	0.1 (0.12)	0.3 (0.23)	0.4 (0.35)	0.2	0.4	0.6

*WL and WH are the low and high limits of spectral width and D is density. Beside them are the mean, standard deviation and median values for the same parameters, converted from the hand drawn notation symbols. Since the spectral widths symbols were drawn along a staff system (logarithmic scale), mean and standard deviation were calculated from midicent values and then converted to Hertz*.

As the standard deviation shows, there were great differences between the actual values from the participants' transcriptions, but the relative patterns of value changes were closer to the original. Answers to the first question (#4), not in Table 3, focused on spectral category. It had sonifications with pitched and dystonic (inharmonic) categories in the sequence: Pitched-Dystonic-Pitched. This was correctly identified by three of the four participants, while one heard the dystonic sound object also as pitched. For next question (#7), concerning the lower limit of spectral width, the mean result is close to the original, though not identical. Two participants had notated the contour correctly. The two following questions (#9 and #12) concern higher limit of spectral width, and here the results have the correct contour but not the correct values–for both questions, three of the four participants had the correct contour. For the last two questions (#14 and #15) involving density changes, the direction of change is correct though not the patterns and values. This was the parameter that was the most difficult to perceive in this freehand study.

## 10. Discussion

The fact that it is possible to identify the data of traditional music notation based on its sonification is not new knowledge. Traditional ear training in music education depends on students abilities to perform such identification with great accuracy. This skill the capacity to identify values and variations in certain music parameters is what Pierre Schaeffer calls qualified perception (Schaeffer, 2017), which is a prerequisite for the comprehension of musical structures based on scales of music parameters.

In the work presented here, we build on this parametric aspect of traditional notation as we sonify spectral parameters from a new system of sound notation. Like central parameters of traditional notation can be represented and sonified using MIDI data, we propose a similar approach for sound notation. Here, both sound and image can be produced from the same numerical data.

Our evaluation, focusing on four music parameters not found in traditional notation, shows that people with some music notation and/or music technology proficiency can identify value changes in these parameters based on their sonification, provided that the participants know what parameter to listen for.

The good performance for the multiple choice survey clearly indicates that the participants could identify the parameter changes in question with an overall mean score of 80% correct answers. The results from the smaller in-depth listening test show that even with freehand transcription, participants could identify patterns or contours of changes for spectral category and spectral width. We believe that training could improve the results from the freehand transcriptions though further testing is needed see to what extent this may be true.

The challenges involved in identifying and transcribing spectral features have to do with the inter-dependence of these parameters. We identified two particular problems: (1) a change in spectral width comes with expectations of change in spectral density and *vice versa* and (2) as the density of a harmonic spectrum decreases, it begins to resemble an inharmonic spectrum causing confusion related to spectrum categories.

What complicates the perception of spectral density is how it is perceived differently for the three spectrum categories. For complex sounds (sounds without pitch) only high spectral densities are possible, or they will turn into inharmonic spectra. The density of a harmonic spectrum is a significant feature for its perception but because of how we perceive multiples of a root frequency together, it is not equivalent to that of an inharmonic spectrum. For harmonic spectra, density is only indirectly experienced. But its effect is nevertheless perceptually important. E.g., a harmonic spectrum with every other partial removed is commonly characterized as having a hollow quality (Helmholtz, 1895).

## 11. Conclusions

This text details our work with sonifying notated complex spectral structures. Listening tests showed that it was possible for listeners with some music training to identify the notation data for individual timbre-related parameters based on their sonification.

While composers sometimes have to accept the simulation of a score as an approximation of the musical performance, e.g., when submitting music to a jury. It is arguably more appropriately used as the sonification of the notated parameters. With this in mind, a system for visual representation and sonification of sound notation was designed—a step toward computer-assisted composition with sound notation adapted from Thoresen's analysis system. As part of this work, a data format similar to MIDI was developed to act as the bridge between the notation and its sonification. This was done in the spirit of Smith's intermediary data format for composing computer music in Music V. Working with additive synthesis turned out to be a convenient solution since it reflects the multi-dimensionality of timbre in which several aspects together inform the characteristics of a single sound (Grey, 1977).

It is important to remember that there can be many ways to change a given spectral aspect of a sound. Spectral density is not only about how densely partials populate the spectral space, it is also about the amplitudes of the partials. Sonifying the spectral centroid is even trickier since it is the center frequency of a spectrum's energy which says little about the amplitudes of any individual partial. Therefore, in the sonification presented here much work went into the design of the synthesis's response to the notated parameters in order to provide relevant sonified feedback from the data.

Future work involves developing automatic analysis of sound structures to provide transcriptions of sound-based music in a software environment. Also, the software has not yet been tested for computer-assisted/algorithmic composition which is an ultimate goal of this research project. There are also several educational uses that will be tested as development of this research proceeds.

## Data Availability Statement

The original contributions presented in the study are included in the article/Supplementary Material, further inquiries can be directed to the corresponding author.

## Ethics Statement

The studies involving human participants were reviewed and approved by KTH Royal Institute of Technology Ethics Officer (Personuppgiftsombud) at: rroy@kth.se. The patients/participants provided their written informed consent to participate in this study.

## Author Contributions

RB: supervised the project and contributed to the editing of the paper. MS: developed the notation system, designed and performed the experiment, and edited the paper.

## Conflict of Interest

The authors declare that the research was conducted in the absence of any commercial or financial relationships that could be construed as a potential conflict of interest.

## Publisher's Note

All claims expressed in this article are solely those of the authors and do not necessarily represent those of their affiliated organizations, or those of the publisher, the editors and the reviewers. Any product that may be evaluated in this article, or claim that may be made by its manufacturer, is not guaranteed or endorsed by the publisher.

## Footnotes

^1^https://www.hauptwerk.com.

^2^Software synthesizers are typically triggered using MIDI note values while cutoff frequency settings affect timbre characteristics.

^3^https://opensoundcontrol.stanford.edu.

^4^https://cycling74.com/products/max.

^5^https://docs.cycling74.com/max8/refpages/ioscbank.

^6^midicents (mc) are used in computer music to make microtonal intervals possible, using MIDI notes as float values.
